# HIV prevalence and high-risk behaviour of young brothel and non-brothel based female sex workers in Nigeria

**DOI:** 10.1186/s13104-017-2712-8

**Published:** 2017-08-10

**Authors:** Uchenna Onyekachi Okafor, Rik Crutzen, Okekearu Ifeanyi, Sylvia Adebajo, Hubertus Van den Borne

**Affiliations:** 1 Department of Health Promotion, Maastricht University, Maastricht, Netherlands; 2 MARPs Division, Society for Family Health, Abuja, Nigeria; 3 HIV AIDs Unit, Population Council International, Abuja, Nigeria

## Abstract

**Background:**

Female sex workers (FSWs) have been identified as a core group in the transmission of HIV and other sexually transmitted infections (STIs). Young FSWs are particularly more vulnerable to HIV due to the combination of vulnerabilities associated with their youth and the sex work they engage in. This study aims to give more insight into HIV prevalence and sexual risk behaviour of young FSWs in Nigeria, by focusing on the differences between BB and NBB young FSWs.

**Methods:**

Data was obtained from the Nigeria Integrated Biological and Behavioural Surveillance Survey (IBBSS) for high-risk groups conducted in 2010. IBBSS is a quantitative survey conducted amongst identified high-risk sub populations within Nigeria. HIV prevalence and risk behaviour data for young BB and NBB FSWs aged 15–24 years for nine states was extracted and analysed.

**Results:**

A total of 1796 FSWs aged 15–24 years were interviewed during the survey, 746 (41.5%) were BB while 1050 (58.5%) were NBB. The HIV prevalence was higher among BB FSWs compared to the NBB FSWs (21.0% vs. 15.5%). BB FSWs reported less condom use with boyfriends and casual partners than NBB FSWs (26.3% vs. 45.5%) and (55.1% vs. 61.1%) respectively while risk of HIV infection due to injecting drug use was higher in NBB compared to BB FSWs (6.6% vs. 1.2%).

**Conclusion:**

Existing and future interventions on HIV prevention should focus on empowering young FSWs with innovative and sustainable approaches aimed at improving their health and wellbeing.

## Background

Young people are a vulnerable sub-population with regard to HIV and related sexual risk behaviours. According to the 2006 population and housing census in Nigeria, young people between 10 and 24 years constitute 31.7% of the 140 million population of the country [1]. In 2010, nearly half of the 3.1 million Nigerians living with HIV were between 15 and 24 years old [2] and young women aged 20–24 bore a high burden with HIV prevalence of 3.2%, very close to the national population prevalence of 3.4% [3]. The findings from the 2012 national HIV/AIDS reproductive and health survey showed that among young persons aged 15–19 years, 37% of the females compared to 20% of the males had engaged in sex with median age at first sex for young women in the rural areas at 15 years compared to 17 years for their male counterparts [3].

Female sex workers (FSWs) have been identified as a core group in the transmission of HIV and other sexually transmitted infections (STIs). Many risk factors contribute to the spread of HIV within FSW networks including their high risk behaviours and sexual practices as well as the high prevalence of STIs [4]. These high risk behaviours and sexual practices include large number of clients, intravenous drug use, excessive alcohol intake, unprotected sex with clients and the existence of non-paying sexual partners; boyfriend and husbands [4, 5]. In addition, the vulnerabilities faced by FSWs relate not only to their individual risk behaviours but also to broader societal and community factors, including gender inequalities, gender based violence, social marginalization, criminalized work environments, limited exposure to social opportunities and health services [6]. These individual and societal factors create a web of vulnerabilities that facilitate the spread of HIV within this population.

Young FSWs are particularly more vulnerable to HIV due to a combination of vulnerabilities associated with their youth and the sex work they engage in. Early sexual debut exposes an individual to the risk of acquiring STIs [7], because the individual is yet to acquire the necessary sex education and social exposure to navigate prevalent gender power imbalances and violence successfully [8, 9]. In Nigeria, societal norms and gender inequalities exist which increase the vulnerabilities of young women to sexual and reproductive health issues compared to their older counterparts [6]. According to a 2013 global review, 35% of women worldwide have experienced either physical and/or sexual intimate partner violence or non-partner sexual violence [10]. The 2013 national demographic and health survey for Nigeria estimates sexual violence against women during their lifetime at 7% and this is more widespread amongst the more vulnerable female population within the society including young FSWs [11].

In Nigeria, typologies of FSWs are differentiated mainly by the location from which their sex work is carried out; brothel based (BB) and non-brothel based (NBB) FSWs. BB FSWs live and work in brothels under the supervision and rules of managers and other power structures existing within the brothel. NBB FSWs include street, home and venue based sex workers who solicit for clients and provide sexual services in streets and public places such as hotels, bars, restaurants and nightclubs. Power structures for NBB FSWs are different and less dependent on the rules and confinement of the brothel owners, pimps and managers [12].

This study aims to provide more insight into HIV prevalence and high-risk behaviour of young FSWs in Nigeria focusing on the differences between BB and NBB FSWs. This is an essential first element of a needs assessment for future HIV prevention interventions [13].

## Methods

This study entailed secondary analysis of data extracted from the Integrated Biological and Behavioural Surveillance Survey (IBBSS) for high-risk groups conducted in 2010 [14]. HIV prevalence and risk behaviour data for young BB and NBB FSWs aged 15–24 years for nine states were analysed.

### Sampling

Eight Nigerian states (Lagos, Kano, Kaduna, Benue, Nasarawa, Edo, Anambra and Cross River) and the Federal Capital Territory (FCT) were selected for the study. To be eligible for recruitment into this study, participants had to be females aged between 15 and 24 years who received money or other valuable gifts/incentives in exchange for sexual favours. A time location sampling (TLS) technique was used to select NBB FSWs. The TLS is a form of cluster sampling that contains both time and location dimensions, it is suitable for obtaining information on hard to reach populations [15]. Working through relevant NGOs and government health staff in different cities/towns, the list of streets, bars, nightclubs and hotels where NBB FSW usually congregate was updated.

The BB FSWs were selected using a two-stage cluster sampling technique. First, a list of brothels where FSW work was drawn and information was collected on the approximate number of FSW present to permit an estimate of cumulative measure of size. Secondly, clusters were selected using probability proportionate to size (PPS) with a fixed number of FSWs recruited from each cluster. The cluster size of the brothel was five and 50 clusters were selected in each state.

### Ethical issues

Participation in the survey was strictly voluntary and no incentives were provided. To guarantee anonymity, no forms of nominal and biometric identifiers were included in the questionnaires. Informed consent was obtained from each participant. Due to the illegal nature of sex work in the country respondents did not want to sign a consent form. Instead, the interviewer explained the study to the participants and obtained verbal consent for the behavioural and biological components of the study and accompanied the participant to the counsellor-tester for HIV tests. All components of the study received ethical approval from the Nigerian Institute for Medical Research’s (NIMR) Institutional Review Board (IRB) in Nigeria and the Family Health International’s (FHI) Protection of Human Subjects Committee.

### Measures

The primary independent variable for the study was the FSW typology: BB or NBB FSWs. Other secondary independent variables include:

#### Highest educational level achieved

Respondents were asked their highest level of education out of seven options—‘never attended school’, ‘Quaranic education’, ‘some primary’, ‘completed primary’, ‘some secondary’, ‘completed secondary education’, and ‘tertiary’ education.

#### Main reason for going into sex work

Ten possible scenarios based on pre-identified circumstances were used to describe this: *‘*financial gain’, ‘unemployment’, ‘pleasure’, ‘marital frustration’, ‘divorced/separated’, ‘widowed’, ‘incest/abused’, ‘others’, ‘don’t know’ and ‘no response’.

#### Clients and duration of sex work

Clients and duration of sex work was measured using the following: how many customers/clients did you have last day worked, last 7 days how many customers/clients did you have, length of time engaged in sex work in months.

Dependent variables were condom use behaviours of interest and HIV prevalence of both populations. The study assessed participant with the following questions and reported those who responded with ‘yes’ or ‘every time’ to any of the following questions:

- Have you had sex with a cohabiting partner within the last 12 months?
- The last time you had sex with a cohabiting partner was condom used?
- Have you had sex with boyfriend within the past 12 months?
- The last time you had sex with boyfriend was a condom used?
- Have you had sex with a casual partner within the last 12 months?
- The last time you had sex with a casual partner was a condom used?
- Within the last 30 days how often did you use condom with your clients?
- Do you charge more for sex without condom?
- Has a client forced you to have sex without condom?
- Are you at risk of HIV infection due to use injectable narcotics?

### Analysis

IBM statistics software SPSS version 20 was used for the extraction and analysis of the data. Descriptive analysis was conducted to characterize the study population of young FSWs (15–24 years). This was followed by bivariate analyses (i.e. Chi square tests and independent samples *t* test) to examine associations between typology of FSW and selected sexual risk behaviour of the FSWs as well as socio-demographic variables.

## Results

A total of 1796 female sex workers comprising BB FSWs (41.5%) and NBB FSWs (58.5%) aged 15–24 years were interviewed during the survey. A higher proportion of NBB FSWs (60.1%) had at least completed secondary education than BB FSWs (35.5%) (p < 0.001) (Table 1).

**Table 1:** Characteristics of NBB and BB FSWs aged 15–24 years

Variables	BB % (n = 746)	NBB % (n = 1050)	X^2^	*p*
Highest educational level achieved				
Never attended school	3.8	1.2	155.1	<0.001
Quaranic education	0.7	0.6		
Some primary	5.6	3.0		
Completed primary	13.0	5.8		
Some secondary	41.4	29.3		
Completed secondary	33.1	44.1		
Tertiary	2.4	16.0		
Main reason for going into sex work				
Financial gain	76.0	82.2	36.5	<0.001
Unemployment	8.7	7.0		
Pleasure	3.4	5.2		
Marital frustration	4.5	2.1		
Divorced/separated	2.1	0.6		
Widowed	0.3	0.3		
Incest/abused	1.4	0.5		
Others	1.8	0.3		
Don’t know	0.3	0.4		
No response	1.6	1.4		

Clients and duration of sex work	BB M (SD)	NBB M (SD)	*t*	*p*
How many customers/clients did you have last day worked	9.6 (19.8)	6.0 (18.8)	3.86	<0.001
Last 7 days how many customers/clients do you have	36.2 (26.1)	16.4 (24.6)	16.29	<0.001
Duration of sex work in months	20.0 (16.6)	21.2 (15.4)	1.55	0.12

Over three-quarters of both BB FSWs and NBB FSWs reported going into sex work for financial gains. When compared to NBB FSWs, BB FSWs reported higher number of clients on last day worked (6 vs. 10) and in the last 7 days (16 vs. 36). However, there was no difference between BB and NBB FSWs in the reported mean duration of sex work: 20.0 (SD 16.6) and 21.2 (SD 15.4) months respectively (p = 0.12).

The HIV prevalence was significantly higher among BB FSWs compared to the NBB FSWs (21.0% vs. 15.5%). A higher percentage of NBB FSWs (45.0%) than BB FSW (26.3%) reported condom use with a cohabiting partner at last sex act. The majority of the respondents in both groups reported condom use during last sex act. BB FSWs reported less condom use with boyfriends (26.3% vs. 45.5%) and casual partners (55.1% vs. 61.1%) than NBB FSWs. However, fewer BB FSWs (4.0%) than NBB FSWs (9.5%) who reported having sex without condoms within the last 30 days charged higher for sex without condom use (p < 0.001). A significantly high proportion of NBB FSWs (17.0%) than BB FSW (7.3%) reported being forced by clients to have sex without condom (p < 0.001). A higher percentage of NBB FSWs (6.6%) than BB FSWs (1.2%) are at risk of HIV due to their use of injectable narcotics (p = 0.002) (Table 2).

**Table 2:** HIV prevalence and sexual risk behaviour of BB and NBB FSWs

Variables	BB %	NBB %	X^2^	*p*
Prevalence of HIV	21.0	15.5	8.68	0.01
Had sex with a cohabiting partner	11.6	10.1	0.94	0.33
Last time you had sex with a cohabiting partner was a condom used	42.4	23.5	8.88	0.01
Had sex with boyfriend within the past 12 months	71.1	76.4	6.43	0.01
Last time you had sex with a boyfriend was a condom used	26.3	45.5	55.81	<0.001
Had sex with a casual partner within the past 12 months	10.8	20.3	28.71	<0.001
Last time had sex with a casual partner was a condom used	55.1	61.1	3.01	0.39
Last time you had sex was a condom used	97.8	93.2	22.45	<0.001
Within the last 30 days how often did you use condoms with your clients	91.1	77.9	59.09	<0.001
Do you charge more for sex without condom	4.0	9.5	23.50	<0.001
Has a client forced you to have sex without condom	7.3	16.9	39.30	<0.001
At risk of HIV due to use of injectable narcotics	1.2	6.6	9.98	0.002

*Note* Boyfriends are non-commercial sexual partners of FSWs who do not reside with the FSW while cohabiting partners are non-commercial sexual partners in a marital or non-marital relationship who reside with the FSW

## Discussion

This study highlights differences in high-risk behaviour and HIV prevalence of BB FSWs and NBB FSWs aged 15–24 years. The number of NBB FSWs within the sampled population was higher than the BB FSWs. This may be attributed to the social stigma associated with living in a brothel and being tagged an FSW. In addition, sex workers are marginalized socially and economically within the society and this makes it undesirable to reside in brothels that are publicly identified as sex work establishments. In addition, most NBB FSWs often times engage in other activities unrelated to sex work and do not want to be tagged openly as sex workers [16].

The daily and weekly number of clients for BB FSWs was higher compared to the NBB FSWs. Risk of HIV and other sexually transmitted infections increases with higher frequency of sexual acts and higher number of sexual partners [11, 17]. The higher number of clients for BB FSWs in this study may have increased their risk to HIV and other sexually transmitted infections. Some of the differences between BB FSWs and NBB FSWs can be ascribed to the environmental and social mechanisms within their work setting as well as socio-demographic indices pertinent to each group. BB FSWs work in more confined settings and often have to pay stipends to the social structures within the brothel system i.e. chairladies and/or brothel owners. Such a system that focuses more on how much money is made than on reducing sexual risk taking by the FSWs promotes HIV and STI risks. Power structures and environmental factors also influence the sexual behavior of FSWs i.e. behavior of pimps, brothel managers and law enforcement agents and this can significantly affect the outcome of interventions targeted at FSWs and their sexual behaviors. The need to target and integrate these structures and factors within prevention interventions addressing the behavior and activities of these environmental agents will enhance the outcome of these interventions [12].

Inconsistent condom use with boyfriends and regular partners observed within this study is consistent with other studies that have reported similar trends by FSWs [4, 8, 17–19]. The higher level of inconsistent condom use by FSWs in brothels may further buttress the need to target them specifically with HIV prevention interventions tailored to address issues specific to their inconsistent condom use which could compromise efforts made thus far in combating the HIV epidemic in Sub-Saharan Africa [20]. Reasons adduced for inconsistent condom use by FSWs with regular partners cite ‘trust’ and ‘feelings of intimacy’ as the primary reasons for lack of condom use as well as the need to create a psychological distinction between their personal and work life [18].

A higher proportion of NBB FSWs reported being forced to have sex without condoms compared to the BB FSWs. The communal structure of the brothels may discourage and reduce incidents of sexual violence within the brothel by partners and/or clients. With the gender inequalities and gender power plays that abound within the society especially among vulnerable groups of women, FSWs are often exposed to sexual violence and rape from intimate-partners and clients. The lack of the communal structures for the NBB FSWs makes them easier preys to sexual violence and also harder to reach with HIV prevention interventions.

Risk of HIV infection due to the use of injectable narcotics exist for both BB and NBB FSWs. Injecting drug use by FSWs increase their risk of HIV infection through the sharing of injecting equipment and the increased likelihood of unprotected sex while under the influence of drugs or experiencing withdrawal [21].

An analysis of the educational attainment of the two groups also shows lower educational attainment for the BB FSWs with a lower percentage of them having completed both secondary and tertiary education compared to their NBB counterparts. Previous studies report an association between low educational attainment, HIV risk perception and sexual behavior [10, 17] especially with regards to self efficacy and assertiveness for consistent condom use.

Financial gain and unemployment were the main reasons cited for going into sex work by both groups thus showing that the prevalent poverty within the society influences the choice of engaging in sex work by FSWs. Many people in Nigeria live in poverty and lack not only money, but also assets and skills. Therefore, in striving to get basic needs they mostly indulge in risky behaviors, such as commercial sex. This is consistent with reports from other studies demonstrating that poverty and economic deprivation make vulnerable women who are without jobs turn to sex work as a means of livelihood to take care of themselves, their families and relatives [17, 22].

## Limitations

A key limitation of this study is the use of self-reported data, which may have resulted in under-reporting of risk behaviour due to social stigma and social desirability bias (i.e. for indices like condom use). Thus the percentage of inconsistent condom use might be even higher than has been shown by this study. Moreover, the hidden nature of the FSW population makes it possible that some of the FSW population in the sampled locations were missed thus making the sampling frame incomplete. However, the sample size for the study and the multiple locations used mitigates the potential impact this might have had on our findings. The study did not focus strongly on the power structures around the FSWs that influence their risk profile within the brothels. These power structures include the brothel owners, chairladies, managers and pimps who largely determine the environment in the brothels within which sex work is carried out.

## Conclusion

FSWs in Nigeria are at high risk of HIV and STIs and are a core group for HIV prevention interventions. Existing and future interventions should focus on empowering young FSWs with innovative and sustainable approaches aimed at improving their health and wellbeing. BB and NBB FSWS are the main FSW subgroups targeted for HIV prevention programs within the country and understanding the dynamics and vulnerabilities within the two groups will enhance intervention quality and ensure activities are tailored to meet their needs and vulnerabilities. BB FSWs show higher risk profiles with regards to number of clients as well as condom use with boyfriends and casual partners within this study. These are important risk factors that should be keenly considered in HIV prevention interventions for this subgroup. The higher HIV prevalence for BB compared to NBB FSWs illustrates the need to incorporate activities and strategies aimed at addressing relevant high-risk behaviours as well as the need to empower them to deal with the environmental and social intricacies and inequalities prevalent within their work place.

Behaviour change interventions designed to positively change high-risk behaviours amongst FSWs should adopt strategies that address identified vulnerabilities at the individual and environmental levels within this group. Power structures within the environment of the FSWs should also be included in prevention interventions and the effect of these prevention focused activities measured and documented.
